# In Memoriam—Dr. Lucien B. Wells

**Published:** 1894-07

**Authors:** 


### IN MEMORIAM—DR. LUCIEN B. WELLS.

The Homœopathic Medical Society of the County of Oneida desires to place upon record and to communicate to his family its estimate of the life, character, and labors in their community of its late associate, Dr. Lucien B. Wells.

In the death of Dr. Wells, this society has lost one of its most earnest and devoted members, and the school of medicine to which he adhered one of its most zealous advocates and defenders; and in common with the other associations to which he belonged, with the church of which for forty years he was a consistent member, and the community in which he had so long lived and labored, it deeply deplores his death.

Decided in his convictions, he was courteous and honorable in all his professional relations and highly esteemed by all who knew him.

Unostentatious and unambitious, he was conscientiously devoted to his profession and faithful in all the varied relations of life.

We shall miss the familiar presence of one who had long passed the period allotted by the psalmist as the usual duration of human life, and whose genial and kindly bearing had endeared him to every member of the profession.

*Resolved,* That we tender to the family of Dr. Wells our most heartfelt sympathy, and that a copy of the minutes be transmitted to them and entered upon the records of the society.

*Resolved,* That this society will attend his funeral in a body.

